# Prehospital ultrasound scanning for abdominal free fluid detection in trauma patients: a systematic review and meta-analysis

**DOI:** 10.1186/s12873-023-00919-2

**Published:** 2024-01-07

**Authors:** Kun-Te Lin, Zih-Yang Lin, Cheng-Chieh Huang, Shang-Yan Yu, Jing-Lan Huang, Jian-Houng Lin, Yan-Ren Lin

**Affiliations:** 1 Department of Emergency and Critical Care Medicine, Changhua Christian Hospital, 135 Nanshsiao Street, 500, Changhua, Taiwan; 2 Department of Biological Science and Technology, National Yang Ming Chiao Tung University, Hsinchu, Taiwan; 3 Fire Bureau of Changhua County Government, Changhua, Taiwan; 4 Department of Post-Baccalaureate Medicine, College of Medicine, National Chung Hsing University, Taichung, Taiwan; 5 School of Medicine, Chung Shan Medical University, Taichung, Taiwan

**Keywords:** Prehospital, Ultrasound, FAST, Trauma

## Abstract

**Introduction:**

Focused assessment with sonography for trauma helps detect abdominal free fluid. Prehospital ultrasound scanning is also important because the early diagnosis of hemoperitoneum may reduce the time to definitive treatment in the hospital. This study investigated whether prehospital ultrasound scanning can help detect abdominal free fluid.

**Materials and methods:**

In this systematic review, relevant databases were searched for studies investigating prehospital ultrasound examinations for abdominal free fluid in trauma patients. The prehospital ultrasound results were compared with computed tomography, surgery, or hospital ultrasound examination data. The pooled sensitivity and specificity values were analyzed using forest plots. The overall predictive power was calculated by the summary receiver operating characteristic curve. The quality of the included studies was assessed using the quality assessment of diagnostic accuracy studies tool. The Grading of Recommendations, Assessment, Development, and Evaluation (GRADE) was performed to assess the certainty of evidence.

**Result:**

This meta-analysis comprised six studies that included 1356 patients. The pooled sensitivity and specificity values were 0.596 (95% confidence interval [CI] = 0.345–0.822) and 0.970 (95% CI = 0.953–0.983), respectively. The pooled area under the summary receiver operating characteristic curve was 0.998. The quality assessment tool showed favorable results. In the GRADE analysis, the quality of evidence was very low for sensitivity and high for specificity when prehospital ultrasound was used for hemoperitoneum diagnosis.

**Conclusion:**

The specificity of abdominal free fluid detection using prehospital ultrasound examinations in trauma patients was very high.

**Supplementary Information:**

The online version contains supplementary material available at 10.1186/s12873-023-00919-2.

## Introduction

Focused assessment with sonography for trauma (FAST) has been widely used in trauma patients to detect free fluid [1]. Compared with computed tomography (CT) or other advanced examinations, ultrasound scanning can be performed at the bedside and on unstable patients, thereby precluding deterioration of patient’s condition during transportation from the scene to examination areas. Additionally, ultrasound helps decision-making and facilitates early diagnosis [2]. The FAST exam serves as an adjunct to primary survey and management in the Advanced Trauma Life Support (ATLS) algorithm [3].

FAST scanning is crucial in abdominal trauma patients because it significantly influences decision-making [4]. In prehospital emergency rescue settings, triage is the process of prioritizing patient treatment during mass-casualty events. Early recognition of critical patients who require emergent management is the top priority. In abdominal trauma patients, any delay in prehospital transport or management of intra-abdominal bleeding can significantly increase the risk of death [5]. Thus, prehospital FAST scanning in abdominal trauma patients is critical, as it allows for the early diagnosis of hemoperitoneum, potentially reducing the time to definitive treatment [6, 7].

However, ultrasound scanning in the prehospital environment poses challenges due to interference in mobile ambulances, insufficient time, difficulty visualizing the screen, the low quality of handheld ultrasound machines, obesity, and abdominal bowel gas distension [8].

Prehospital ultrasound has been widely used in various situations. For instance, prehospital ultrasound chest scans help diagnose pneumothorax and hemothorax [9, 10]. In out-of-hospital cardiac arrest patients, prehospital ultrasound can detect cardiac activity during resuscitation [11, 12]. Despite its usefulness in these scenarios, prehospital ultrasound scanning for abdominal free fluid detection has not been well evaluated, and a detailed meta-analysis is lacking. Therefore, this study aimed to investigate whether prehospital ultrasound scanning can also help detect abdominal free fluid in trauma patients.

## Materials and methods

### The study protocol and literature search strategy

This study was performed according to the preferred reporting items for systematic review and meta-analysis (PRISMA) guidelines [13]. A completed preferred reporting item for systematic review and meta-analysis protocols (PRISMA-P) checklist was provided in supplemental appendix file 1. Electronic searches were performed by two authors (Kun-Te Lin and Cheng-Chieh Huang) using the PubMed and Embase databases, the Cochrane Central Register of Controlled Trials, and the Cochrane Database of Systematic Reviews up to August 3, 2023. The search terms used were “prehospital” AND (“ultrasound” OR “echography” OR “focused assessment with sonography for trauma” OR “FAST”) AND “trauma”. Search strategies for all databases were listed in supplemental appendix file 2.

The inclusion criteria were as follows:

1. Randomized or observational trials in trauma patients who received a free fluid exam involving a prehospital abdominal ultrasound.
2. Patients who underwent abdominal ultrasound examinations, CT examinations, or surgery in the hospital; these patients were used as the reference standard for abdominal free fluid evaluation.

The exclusion criteria were as follows:

1. Articles without full text available or that were not written in English.
2. Case series studies, conference papers, or studies without recorded data.

### Data synthesis and statistical analysis

We recorded the first author, year, study design, clinical scenario, patient number, index test, and reference test for each study. True positive (TP), true negative (TN), false positive (FP), and false negative (FN) values, sensitivities, and specificities were extracted from each study for prehospital abdominal ultrasound in free fluid examinations. A random-effects model was employed to pool the sensitivities and specificities of prehospital ultrasound examinations with their corresponding 95% confidence intervals (CI). The pooled results were presented using forest plots. Additionally, the overall predictive power was calculated by constructing a summary receiver operating characteristic (sROC) curve. The heterogeneity between the studies was determined by a chi-square test. Values over 50% were considered to have considerable heterogeneity [14]. Furthermore, a funnel plot was used to examine potential publication bias [15]. The statistical analyses were performed using Review Manager (Version 5.4.1, The Cochrane Collaboration, The Nordic Cochrane Centre, Copenhagen, Denmark).

### Bias and study quality assessment

The Quality Assessment of Diagnostic Accuracy Studies (QUADAS-2) tool was used to assess the bias and quality of the included studies [16]. Two main categories were evaluated: the risk of bias and applicability concerns. The tool comprised four main domains: patient selection, index test, reference standard, and flow and timing. Each domain was categorized as low-risk (green), unclear risk (yellow), or high-risk (red). Any discrepancies between reviewers were resolved through discussions by two authors (Kun-Te Lin and Zih-Yang Lin).

### Assessment of evidence certainty

The Grading of Recommendations, Assessments, Development, and Evaluations (GRADE) was utilized to evaluate the certainty of evidence in this meta-analysis [17]. This evaluation was carried out by two authors (Kun-Te Lin and Zih-Yang Lin). In GRADE, the certainty of evidence was assessed based on five domains: risk of bias, indirectness, inconsistency, imprecision, and publication bias. The test accuracy for sensitivity and specificity was then categorized into four levels of evidence: “very low”, “low”, “moderate”, and “high”.

## Result

### Literature review

**Fig. 1:**
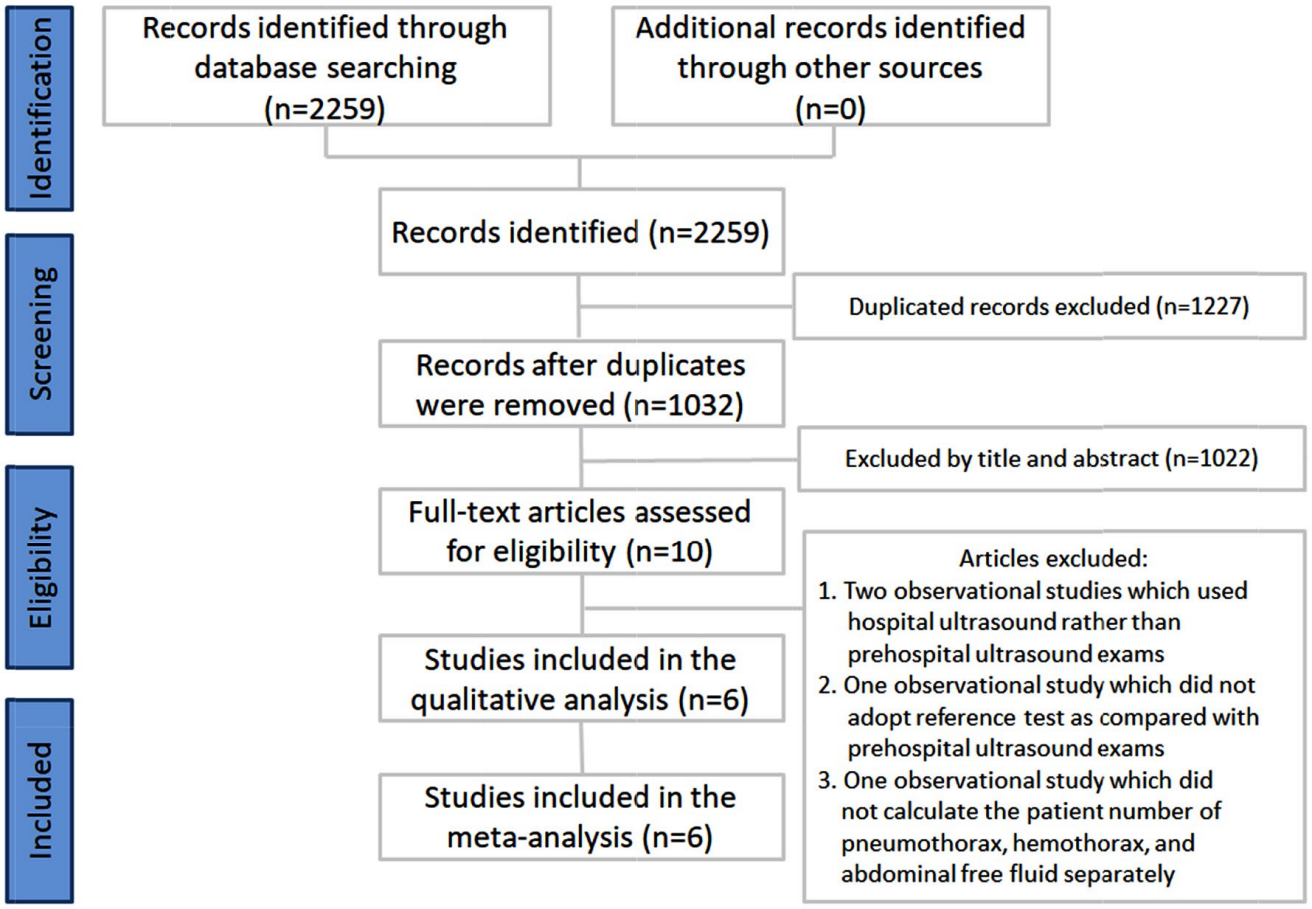
Inclusion process of the selected studies

A total of 2259 publications were identified through electronic searches and literature review. After duplicate removal, 1032 publications were selected for meticulous evaluation. Ten publications were selected for full-text retrieval after title and abstract evaluations. The following four studies were excluded from the analysis: two observational studies [18, 19] that investigated hospital rather than prehospital ultrasound examinations, one observational study [20] that did not adopt a reference test in comparing ultrasound findings, and one observational study [10] that did not involve the independent quantification of patients’ number of pneumothorax, hemothorax, and abdominal free fluid. Finally, six studies were used for qualitative analysis and meta-analysis [21–26]. The literature review flow chart is shown in Fig. 1. The patient numbers and characteristics of the included studies are listed in Tables 1 and 2.

**Table 1:** Characteristics of the included studies

Author/year	Study design	Situation	Number of patients analyzed				Method	Target condition	Reference test
			H+		H-				
			P+	P-	P+	P-			
Walcher et al. 2006	Prospective observational	Trauma	26	2	1	173	PFAST	Abdominal free fluid	ED ultrasound or CT
Gregory et al. 2014	Prospective observational	Trauma	12	14	10	161	PFAST	Abdominal free fluid	CT or surgery
Ketelaars et al. 2019	Retrospective observational	Trauma	28	64	10	319	Prehospital ultrasound	Abdominal free fluid	CT or surgery
Gamberini et al. 2022	Retrospective observational	Trauma	17	10	0	17	PFAST	Abdominal free fluid	FAST, CT, or surgery
Lucas et al. 2022	Prospective randomized trial	Trauma	18	1	3	120	PFAST	Abdominal free fluid	CT
Partyka et al. 2022	Retrospective observational	Trauma	21	63	9	257	PFAST	Abdominal free fluid	CT or surgery

PFAST, prehospital focused assessment with sonography for trauma; CT, computed tomography; ED, emergency department; H+, hemoperitoneum was noted in hospital; H-, no hemoperitoneum was noted in hospital; P+, hemoperitoneum was noted on prehospital ultrasound exam; P-, no hemoperitoneum was noted on prehospital ultrasound exam

**Table 2:** Summary of the prehospital ultrasound exam scenarios

Author/year	Setting	Operator	Machine
Walcher et al. 2006	On the scene	Doctor or paramedics	PRIMEDIC™ HandyScan
Gregory et al. 2014	In-flight	HEMS flight nurses or paramedics	M-turbo, Fujifilm Sonosite
Ketelaars et al. 2019	On the scene or during transport	HEMS physician	Fujifilm Sonosite 1. MicroMaxx 2. NanoMaxx 3. M-Turbo
Gamberini et al. 2022	On the scene	EMS	(1) NanoMaxx, Fujifilm Sonosite (2) Vscan Extent™
Lucas et al. 2022	On the scene	Physician	PRIMEDIC™ HandyScan
Partyka et al. 2022	On the scene	Physician	M-turbo, Fujifilm Sonosite

HEMS, helicopter emergency medical services; EMS, emergency medical services

### Pooled analysis of the included studies

**Fig. 2:**
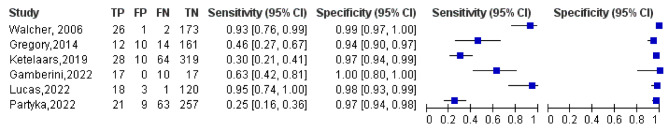
Sensitivity and specificity of the selected studies

**Fig. 3:**
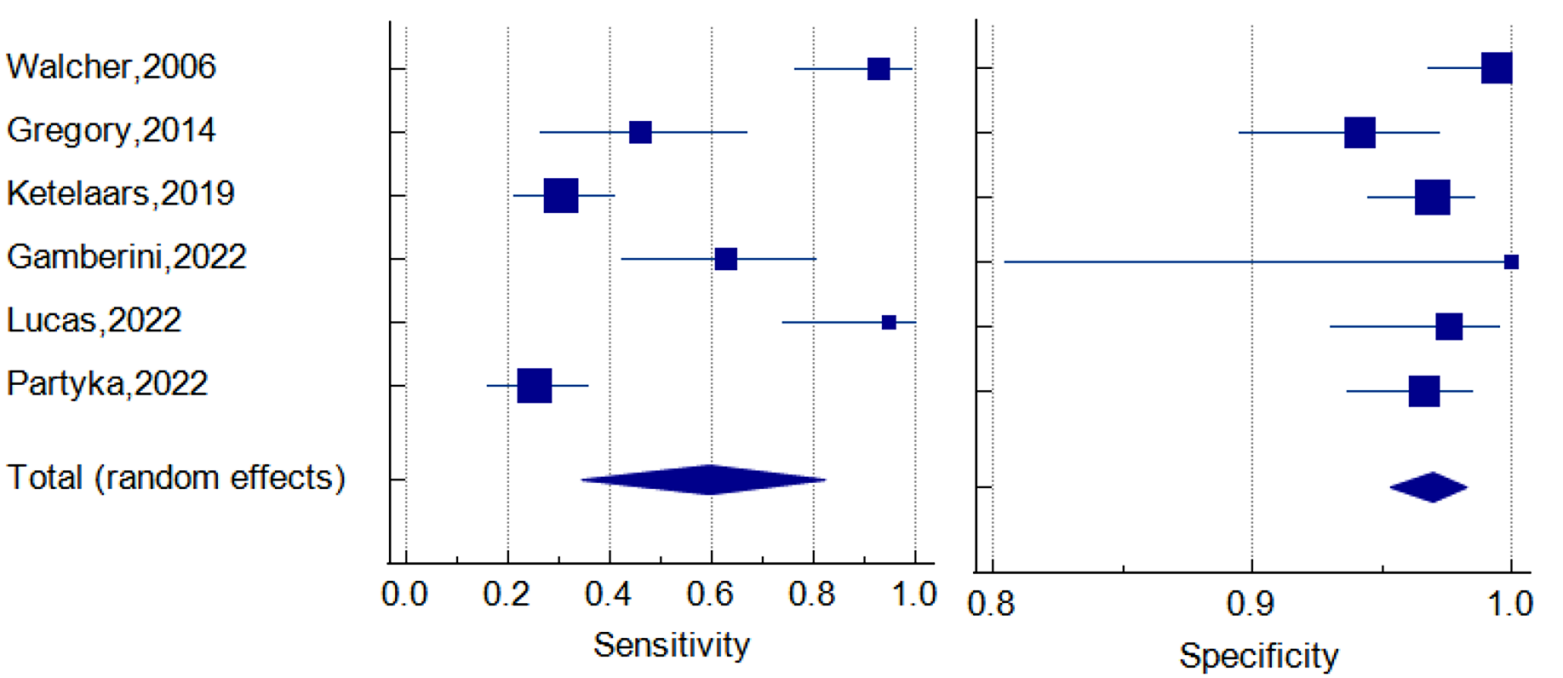
Forest plots of pooled sensitivity and specificity values

**Fig. 4:**
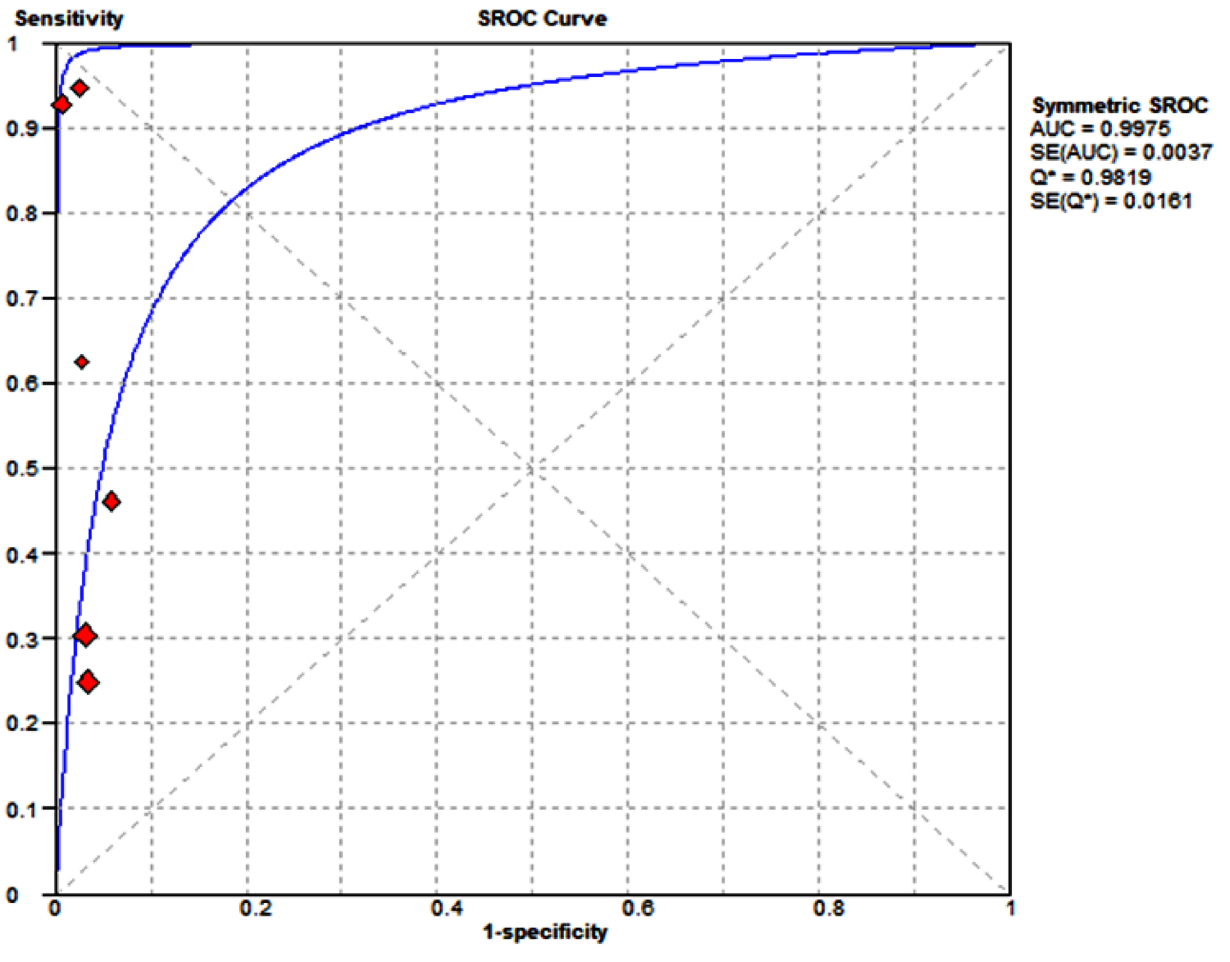
The sROC curve

**Fig. 5:**
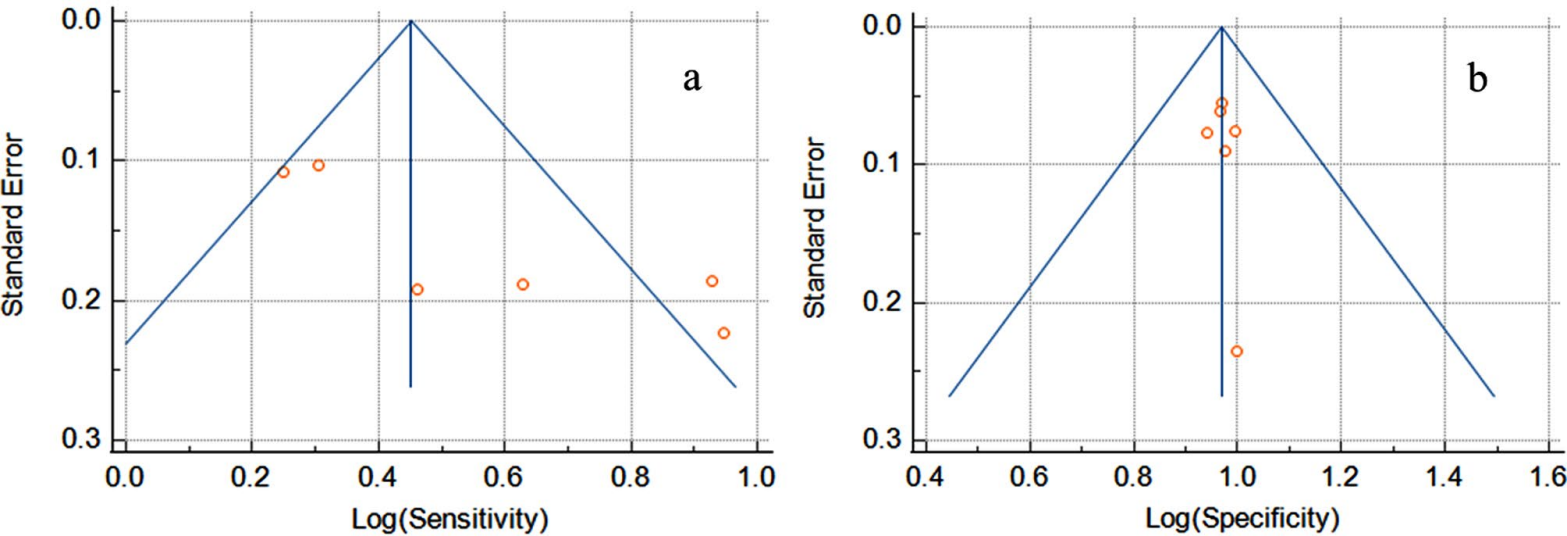
Funnel plots of sensitivity and specificity

A total of 1356 patients from six studies were included in this meta-analysis. The forest plot of pooled sensitivity was 0.596 (95% CI = 0.345–0.822), while the pooled specificity was 0.970 (95% CI = 0.953–0.983), as illustrated in Figs. 2 and 3, respectively. The sROC curve is displayed in Fig. 4. The pooled area under the curve (AUC) of the sROC curve was 0.998. The heterogeneity of sensitivity was 94%, and the heterogeneity of specificity was 45%, both determined by the chi-square test. The funnel plots of sensitivity and specificity are shown in Fig. 5a and b.

### Quality assessment of the selected studies and certainty of evidence for the meta-analysis

**Fig. 6:**
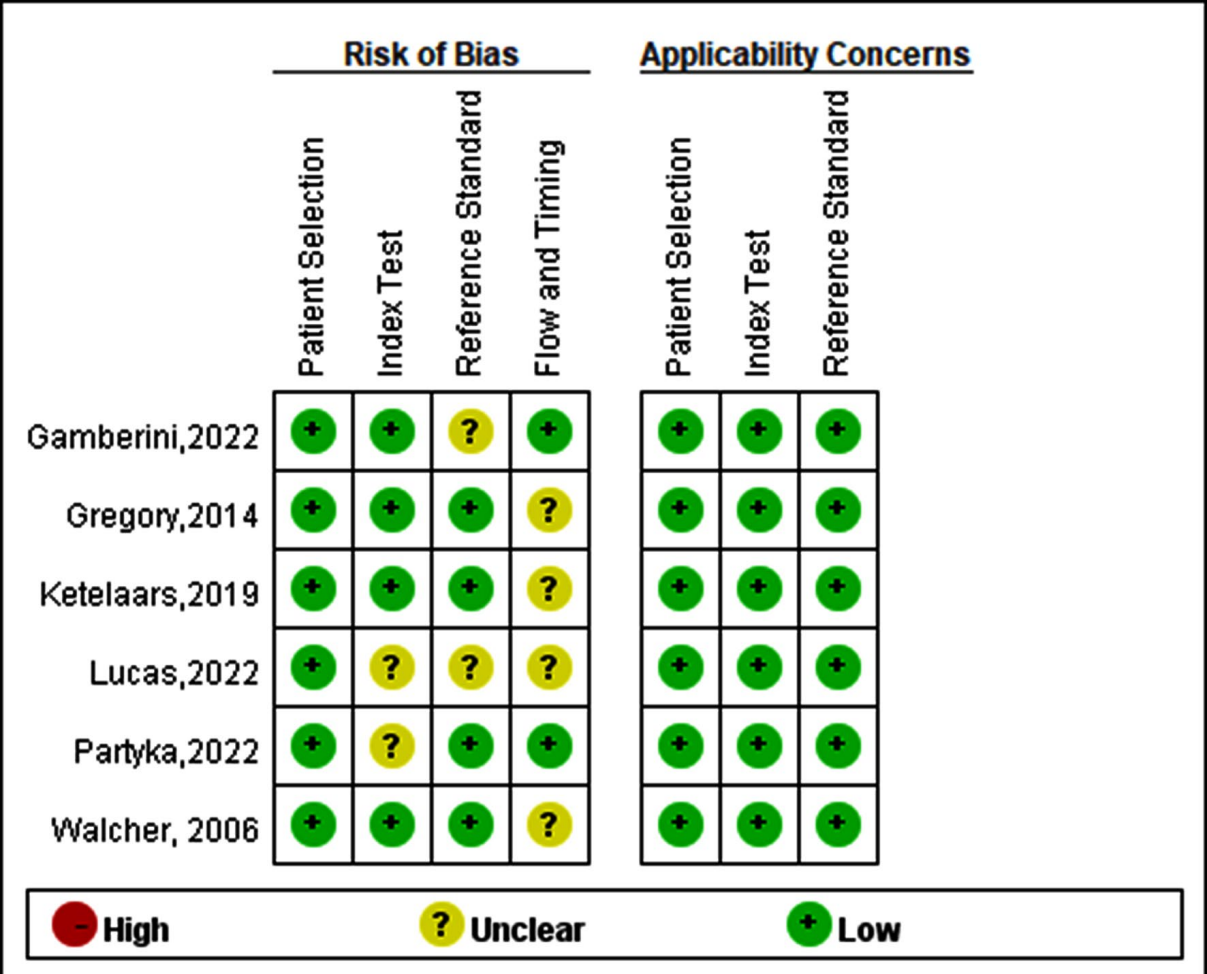
Quality assessment and risk of bias in the included studies

The risk of bias analysis and quality assessment of the included studies are presented in Fig. 6; these evaluations were conducted using the QUADAS-2 tool. Overall, the risk of bias was low, and the clinical applicability was suitable in most of the selected studies. Each study achieved at least four out of seven low-risk bias point assessments. Regarding the certainty of evidence for the meta-analysis, it was rated as very low for sensitivity and high for specificity using the GRADE approach evaluation. The detailed GRADE evaluation result is shown in Table 3.

**Table 3:** The grading of recommendations, assessment, development, and evaluation assessment

Question: Should Prehospital ultrasound be used to diagnose hemoperitoneum?							
Outcome	№ of studies (№ of patients)	Factors that may decrease certainty of evidence					Test accuracy CoE
		Risk of bias	Indirectness	Inconsistency	Imprecision	Publication bias	
True positives (patients with hemoperitoneum)	6 studies 276 patients	Not serious	Not serious	Serious^a^	Serious^b^	Publication bias strongly suspected^c^	⨁◯◯◯ Very low
False negatives (patients incorrectly classified as not having hemoperitoneum)							
True negatives (patients without hemoperitoneum)	6 studies 1080 patients	Not serious	Not serious	Not serious	Not serious	None	⨁⨁⨁⨁ High
False positives (patients incorrectly classified as having hemoperitoneum)							

^a^High heterogeneity for sensitivity between studies, determined by chi-square test was 94%

^b^Wide confidence interval for sensitivity

^c^The funnel plot of sensitivity was asymmetrical

## Discussion

In this meta-analysis of prehospital ultrasound for the diagnosis of abdominal free fluid in trauma patients, the pooled sensitivity and specificity values were 0.596 and 0.970, respectively. Although the pooled sensitivity was not high, the pooled specificity was highly favorable. The funnel plot of sensitivity showed asymmetry, indicating the presence of publication bias for sensitivity. However, the funnel plot of specificity displayed a mostly centralized distributed, suggesting no significant publication bias for specificity. The heterogeneity between the studies was identified in the pooled sensitivity analysis and by visualizing the sROC curve. The heterogeneity revealed that different ultrasound operators, physicians, or paramedics performing prehospital ultrasound scanning could have variable sensitivity and specificity results. The AUC of the sROC curve was 0.998, indicating excellent diagnostic performance [27] of prehospital ultrasound scanning in evaluating abdominal free fluid.

The insufficient sensitivity of prehospital ultrasound scanning for identifying abdominal free fluid may be attributed to the following reasons. First, ultrasound is operator dependent, and it takes time to perform suitable scanning. The challenging conditions inside mobile ambulances and helicopters may further impede the ability to quickly obtain a perfect view during prehospital scanning. Second, ultrasound is less sensitive in detecting retroperitoneal free fluid compared to CT and surgical intervention, which can identify retroperitoneal free fluid more readily [4, 28, 29]. Third, the time interval between prehospital ultrasound scanning and hospital CT scan or surgery may contribute to the discrepancy in sensitivity. The initial level of hemoperitoneum may be insufficient to yield a positive finding during prehospital ultrasound scanning. As time progresses, the accumulation of hemoperitoneum could become visible on subsequent examinations [30, 31].

In the interpretation of prehospital ultrasound screening for abdominal free fluid, a positive finding suggests the presence of hemoperitoneum, but a negative finding should not be used definitely exclude its existence. Given the potential challenges and limitations of prehospital ultrasound scanning, a second look and follow-up exam should be considered, especially in cases where there is high clinical suspicion of abdominal free fluid.

The quality assessment and risk of bias using the QUADAS-2 tool showed a favorable overall quality assessment of the included studies. A low risk of bias was observed in at least four out of seven assessments per study. The flow and timing assessment was mainly attributed to unclear risks of bias in selected studies because the time between the prehospital ultrasound scan and the CT scan or surgery in the hospital was not clearly recorded. As a result, the certainty of the flow and timing and its potential impact on the diagnostic accuracy might be less robust. However, the applicability concerns were all rated as low risk because the patients chosen from each study matched the inclusion criteria of this meta-analysis.

The certainty of evidence for the meta-analysis was rated as very low for sensitivity and high for specificity using the GRADE approach evaluation. The low certainty of evidence for sensitivity might be attributed to factors such as variations in ultrasound operators, different environment conditions, different patient characteristics, and diverse ultrasound machines. These factors could introduce heterogeneity in the sensitivity results, making it challenging to draw firm conclusions. On the other hand, the high certainty of evidence for specificity suggests that the finding of high specificity in prehospital ultrasound scanning for detecting hemoperitoneum is reliable and consistent across the included studies. This indicates that if hemoperitoneum is detected using prehospital ultrasound, it is highly likely to be present, making it a valuable tool in the assessment of trauma patients.

The application of prehospital ultrasound scanning in trauma has recently increased [32]. Prehospital ultrasound scanning is helpful in mass casualty incident triage [33, 34]. Early diagnosis of hemoperitoneum using prehospital ultrasound scanning shortens the time to definitive management and facilitates timely therapy plan changes [6, 7]. A previous study found that a positive prehospital FAST result was associated with a significantly reduced time to definitive management compared to a negative result (20 min vs. 138 min) [35]. Prehospital ultrasound examinations also influenced important treatment decisions related to patients’ information provided to the receiving hospital, the method of transport, the choice of destination hospital, and fluid management approaches in 12.6% of patients in a retrospective analysis [23].

## Conclusion

In this meta-analysis, prehospital ultrasound scanning demonstrated high specificity in the statistical analyses of traumatic abdominal free fluid evaluations. The high AUC of the sROC curve indicated good diagnostic performance of prehospital ultrasound scanning for abdominal free fluid detection. The highly specific but not highly sensitive result implies that that that a positive ultrasound finding suggests the presence of hemoperitoneum, but a negative result does not exclude its presence entirely. Therefore, a second look or follow-up exam should be considered.

This meta-analysis still had some limitations. First, heterogeneity existed between the included studies, indicating that different operators, physicians, or paramedics performing prehospital ultrasound scanning could have influenced the sensitivity and specificity results. Second, the different interval timing between prehospital ultrasound scanning and CT scan or surgery in the hospital may have interfered with the accurate detection of hemoperitoneum. Regretfully, the included studies did not record the interval timing clearly. Future studies should prioritize investigating and standardizing the interval timing to better understand the diagnostic capabilities of prehospital ultrasound in detecting initial hemoperitoneum.

### Electronic supplementary material

Below is the link to the electronic supplementary material.

Supplementary Material 1: PRISMA-P check list

Supplementary Material 2: Search strategies

## Data Availability

The datasets used and/or analyzed during the current study are available from the corresponding author on reasonable request.
