# Supplementary material for: Prehospital ultrasound scanning for abdominal free fluid detection in trauma patients: a systematic review and meta-analysis
Source: BMC Emerg Med. 2024 Jan 7;24:7. doi: 10.1186/s12873-023-00919-2 (PMC10773115; doi:10.1186/s12873-023-00919-2)
Supplement: Supplementary file 2 — Supplementary Material 2: Search strategies [file 12873_2023_919_MOESM2_ESM.pdf]

Appendix 2: Search strategy for databases of Pubmed/Embase/Cochrane Central Register of Controlled Trials, and the Cochrane Database of Systematic Reviews

	**Search terms**
#1	Prehospital
#2	Ultrasound
#3	Echography
#4	Focused assessment with sonography in trauma
#5	FAST
#6	#2 OR #3 OR #4 OR #5
#7	Trauma
#8	#1 AND #6 AND #7
#9	Randomized controlled trial
#10	Clinical trial
#11	#9 OR #10
#12	#8 AND #11
